# Fluvoxamine: A Review of Its Mechanism of Action and Its Role in COVID-19

**DOI:** 10.3389/fphar.2021.652688

**Published:** 2021-04-20

**Authors:** Vikas P. Sukhatme, Angela M. Reiersen, Sharat J. Vayttaden, Vidula V. Sukhatme

**Affiliations:** ^1^ Department of Medicine and the Morningside Center for Innovative and Affordable Medicine, School of Medicine, Emory University, Atlanta, GA, United States; ^2^ Department of Psychiatry, School of Medicine, Washington University in St. Louis, St. Louis, MO, United States; ^3^ Independent Researcher, Montgomery Village, MD, United States; ^4^ GlobalCures, Inc., Newton, MA, United States; ^5^ Department of Epidemiology, Rollins School of Public Health, Emory University, Atlanta, GA, United States

**Keywords:** SARS-CoV-2, cytokine storm, acute respiratory distress syndrome, interleukins, inflammation

## Abstract

Fluvoxamine is a well-tolerated, widely available, inexpensive selective serotonin reuptake inhibitor that has been shown in a small, double-blind, placebo-controlled, randomized study to prevent clinical deterioration of patients with mild coronavirus disease 2019 (COVID-19). Fluvoxamine is also an agonist for the sigma-1 receptor, through which it controls inflammation. We review here a body of literature that shows important mechanisms of action of fluvoxamine and other SSRIs that could play a role in COVID-19 treatment. These effects include: reduction in platelet aggregation, decreased mast cell degranulation, interference with endolysosomal viral trafficking, regulation of inositol-requiring enzyme 1α-driven inflammation and increased melatonin levels, which collectively have a direct antiviral effect, regulate coagulopathy or mitigate cytokine storm, which are known hallmarks of severe COVID-19.

## Introduction

**FIGURE 1:**
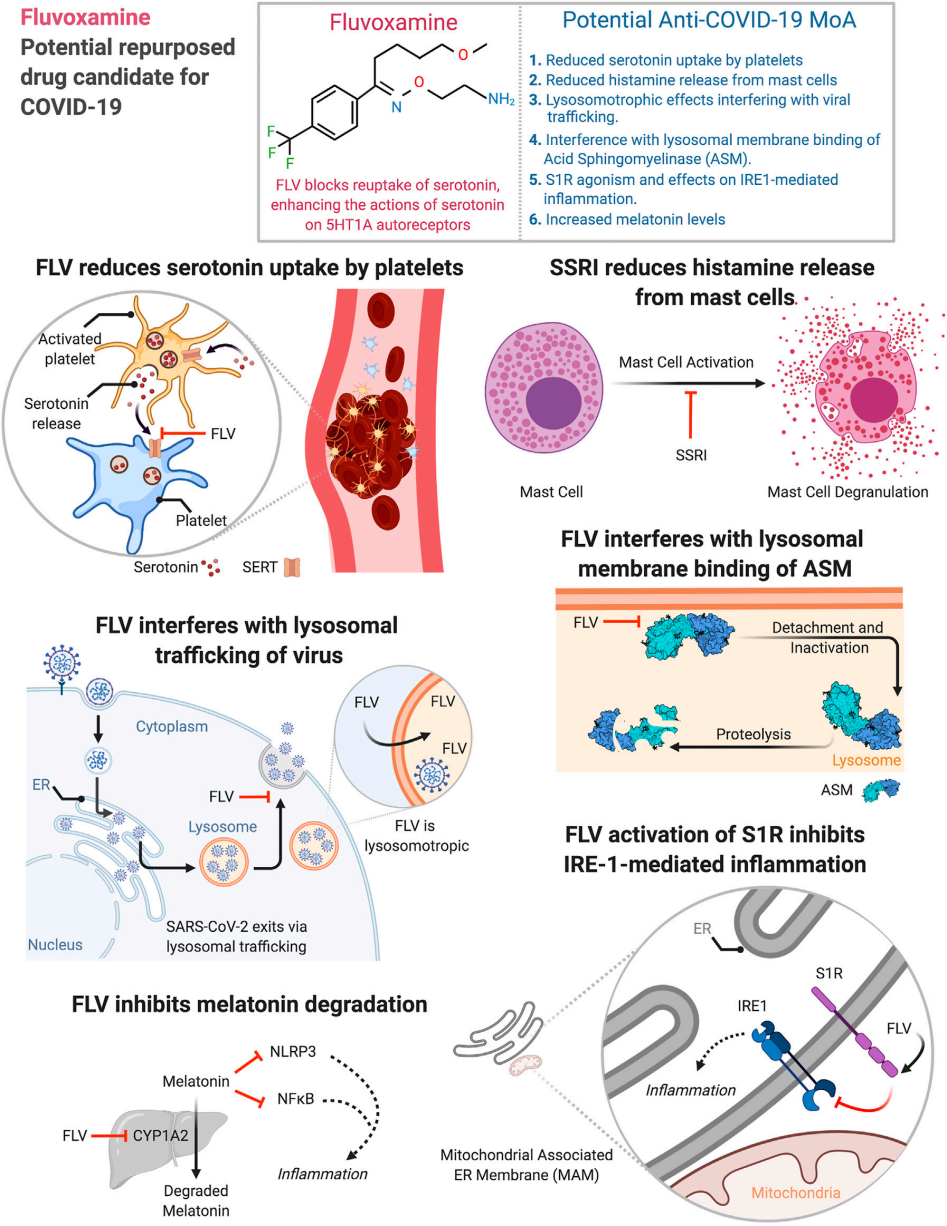
Potential anti-COVID-19 mechanisms of action of fluvoxamine. Figure created using Biorender.

Initially used to treat obsessive-compulsive disorder (OCD), fluvoxamine (FLV) has been shown to have the strongest activity of all SSRIs at the sigma-1 receptor (S1R) with low-nanomolar affinity (Narita et al., 1996). FLV agonism on S1R potentiates nerve-growth factor (NGF)-induced neurite outgrowth in PC 12 cells (Nishimura et al., 2008; Ishima et al., 2014). S1R is a chaperone protein at the endoplasmic reticulum with anti-inflammatory properties (Ghareghani et al., 2017). FLV’s anti-inflammatory effects likely stem from its regulation of S1R, which modulates innate and adaptive immune responses (Szabo et al., 2014). S1R is also an important regulator of inositol-requiring enzyme 1α (IRE1)-driven inflammation (Rosen et al., 2019) (Figure 1).

FLV and other SSRIs regulate inflammatory cytokine activity and gene expression in both cell and animal models of inflammation (Taler et al., 2007; Tynan et al., 2012; Rafiee et al., 2016; Ghareghani et al., 2017; Naji Esfahani et al., 2019; Rosen et al., 2019). The potential of FLV to dampen cytokine storm has implications in COVID-19. COVID-19 severity is associated with an increased level of inflammatory mediators including cytokines and chemokines (Chen G. et al., 2020; Chen N. et al., 2020; Huang et al., 2020; Tay et al., 2020). Other S1R agonists like fluoxetine have been reported to have antiviral activity (Zuo et al., 2012; Bauer et al., 2019). These studies have raised interest in the potential therapeutic role of FLV and S1R agonists in COVID-19 (Vela, 2020; Hashimoto, 2021).

This review illustrates mechanisms of action underlying anti-inflammatory and antiviral properties of FLV. It covers preclinical studies on effects of FLV and S1R agonists on inflammation, and summarizes currently available clinical data for FLV treatment in COVID-19.

### Indications for Fluvoxamine

Fluvoxamine maleate is available as immediate release tablets and controlled-release capsules. FLV is indicated to treat obsessions and compulsions in patients with OCD. The half-life of FLV is 9–28 h depending on its formulation, and the recommended dosage is 100–300 mg/day (FDA, 2012).

## Original Mechanism of Action

### Serotonin Transporter Inhibition

FLV blocks reuptake of serotonin at the sodium-dependent serotonin transporter (SERT) of the neuronal membrane, enhancing actions of serotonin on 5HT1A autoreceptors (Dell'Osso et al., 2005; FDA, 2012). FLV has negligible affinity for α1-, α2-, β-adrenergic, muscarinic, dopamine D2, histamine H1, GABA-benzodiazepine, opiate, 5-HT1, or 5-HT2 receptors (Irons, 2005).

## Likely Mechanisms of Action in COVID-19

### Platelet Aggregation

Platelets lack the enzyme to synthesize serotonin (Ni and Watts, 2006). A SERT enables rapid uptake of serotonin from plasma (Vanhoutte, 1991). During thrombosis platelets release serotonin, facilitating hemostasis through platelet aggregation (Berger et al., 2009) (Figure 1), and promotes recruitment of neutrophils (Duerschmied et al., 2013). SSRIs can therefore increase bleeding time (Leung and Shore, 1996) or reduce serum serotonin by >80% and reduce neutrophil recruitment (Duerschmied et al., 2013). Platelets from individuals treated with SSRIs, and platelets from SERT knockout mice, show decreased aggregation (Celada et al., 1992; Carneiro et al., 2008; McCloskey et al., 2008). Measures of coagulation and hemostasis were lower in patients with serotonergic antidepressant than in patients without serotonergic antidepressant (Geiser et al., 2011). A hyperserotonergic state distinguishes COVID-19 and non-COVID-19 acute respiratory distress syndrome, biochemically (Zaid et al., 2021) and clinically (Helms et al., 2020a; Helms et al., 2020b). This is likely pathologic across a multitude of organs (akin to serotonin syndrome, F. Jalali—personal observation and communication) and may originate from an immune-mediated (Althaus et al., 2020; Nazy et al., 2021) state of platelet hyperreactivity (Zaid et al., 2021), resulting in florid platelet degranulation of serotonin into plasma.

A concomitant impairment of serotonin reuptake may exacerbate this hyperserotonergic state. Serotonin clearance relies on a *healthy* pulmonary endothelium (Thomas and Vane, 1967; Joseph et al., 2013), that is injured in COVID-19 (Ackermann et al., 2020). Platelet serotonin liberation can be reduced with *chronic* or *early de novo* SSRI use (Cloutier et al., 2018), since SSRIs deplete serotonin content of platelets (Narayan et al., 1998; Javors et al., 2000). Initiation of *de novo* SSRIs at *later* stages of moderate to severe COVID-19, however, may be unpredictably harmful given the existing hyperserotonergic state (Zaid et al., 2021) unless counterbalanced by other beneficial effects of SSRIs. Indeed, direct serotonin antagonism specifically targeting the serotonin 2 A, B and C receptors with drugs such as cyproheptadine or mirtazapine in this stage may be beneficial and is being explored (F. Jalali—personal communication).

Three trials assessing benefit of anticoagulants to treat COVID-19 have paused enrollment of critically ill COVID-19 patients who require intensive care unit (ICU) support (NHLBI, 2020). Therapeutic blood thinners did not reduce need for ICU admission in this patient-group. Since full doses of therapeutic anticoagulants increase risk of internal bleeding, FLV could perhaps inhibit blood clotting more safely.

### Mast Cell Degranulation

Human mast cells (MCs) are a viral reservoir for RNA viruses like HIV (Sundstrom et al., 2004). Retinoic acid-inducible gene-I-like receptors of mast cells can detect RNA viruses (Fukuda et al., 2013). Viruses can cause degranulation of MCs in a Sphingosine-1-Phosphate (S1P) -dependent pathway (Wang et al., 2012). MCs express angiotensin converting enzyme 2 (ACE2), the principal receptor for SARS-CoV-2 entry into cells, thus defining a route by which MCs could become hosts for this virus (Theoharides, 2020). Post-mortem lung biopsies of COVID-19 patients have linked pulmonary edema and thromboses to activated MCs (Motta Junior et al., 2020). Antidepressants also decrease histamine release from MCs (Ferjan and Erjavec, 1996). SSRIs like fluoxetine decreased mRNA levels of protease-1 in MCs (Chen et al., 2008). Therefore, SSRIs like FLV could reduce cytokine storms in COVID-19 patients (Figure 1) because of atypical response of MCs to SARS-CoV-2.

### Lysosomotropism

S1R agonists like FLV and fluoxetine are lysosomotropic (Hallifax and Houston, 2007; Kazmi et al., 2013). Fluvoxamine has a predicted pKa of 8.86 (DrugBank, 2005; Wishart et al., 2018) and is susceptible to protonation in the physiological pH range. Less polar, unionized form of basic drugs can easily cross membranes. Basic drugs like FLV can get protonated in the lysosome, which hinders the now-charged moieties from crossing membranes. β-coronaviruses, like SARS-CoV-2 and mouse hepatitis virus (MHV), use lysosomal trafficking to escape from infected cells (Ghosh et al., 2020) (Figure 1). GRP78/BIP, a chaperone that facilitates coronavirus infectivity (Chu et al., 2018; Ha et al., 2020), is co-released with β-coronaviruses through this pathway (Ghosh et al., 2020). The SARS-CoV open reading frame protein 3A (ORF3a) (Gordon et al., 2020) is a viroporin that localizes to lysosomes (Ghosh et al., 2020), disrupts their acidification (Yue et al., 2018), and contributes to viral egress (Lu et al., 2006; Castano-Rodriguez et al., 2018; Yue et al., 2018). Given the lysosomal egress of β-coronaviruses from infected cells, lysosomotropic drugs like FLV could have antiviral effects in the virus laden lysosomes (Homolak and Kodvanj, 2020) (Figure 1).

### Acid Sphingomyelinase

Lysosomotropic drugs displace acid sphingomyelinase (ASM) from lysosomal membranes leading to its degradation (Breiden and Sandhoff, 2019) (Figure 1). Treatment of mice with S1R agonists like fluoxetine (Hashimoto, 2015) reduces both acid sphingomyelinase activity and protein levels in neurons (Gulbins et al., 2013). This is consistent with partial proteolysis of acid sphingomyelinase by fluoxetine (Kornhuber et al., 2008). Fluoxetine can efficiently inhibit entry and propagation of SARS-CoV-2 in Vero-E6 cell lines (Schloer et al., 2020). It also exerts antiviral activity against influenza A virus subtypes (Schloer et al., 2020). S1R agonists like escitalopram and fluoxetine (Hashimoto, 2015) can prevent infection of Vero cells with vesicular stomatitis virus pseudoviral particles presenting SARS-CoV-2 spike protein (pp-VSV-SARS-CoV-2 spike) (Carpinteiro et al., 2020). Antidepressants like amitriptyline also prevented infection of human Caco-2 cells with SARS-CoV-2 and treating volunteers with a low dose of amitriptyline prevented infection of freshly isolated nasal epithelial cells with pp-VSV-SARS-CoV-2 spike (Carpinteiro et al., 2020). Inhibition of acid sphingomyelase by these drugs can prevent the conversion of sphingomyelin to phosphorylcholine and ceramide. Because high ceramide in the cell membrane facilitates viral entry, this reduction in ceramide may prevent infection (Carpenteiro et al., 2020). Therefore, functional inhibition of acid sphingomyelinase by lysosomotropic drugs is another avenue of viral control by antidepressants.

### Sigma-1 Receptor Activity

S1R was discovered in 1976 (Martin et al., 1976) and cloned in 1996 (Hanner et al., 1996). It regulates ER-mitochondrial Ca^2+^ signaling and cell survival (Hayashi and Su 2007). Targeting S1R with FLV regulates cytokine production in human monocyte-derived dendritic cells (Szabo et al., 2014). S1R knockout (KO) bone marrow-derived macrophages (BMDMs) were proinflammatory in endotoxic shock models. They had higher levels of IL-6 and IL-1*β* mRNA and increased IL-6 protein secretion compared to wild-type (WT) BMDMs (Rosen et al., 2019). In contrast, anti-inflammatory cytokine IL-10 expression was unaffected in S1R KO BMDMs (Rosen et al., 2019). S1R overexpression in HEKs expressing mTLR4/MD2/CD14 was anti-inflammatory in an endotoxic shock model. Compared to HEKs with normal levels of S1R, cells with higher levels of S1R had lower IL-8 levels on LPS stimulation (*p* < 0.05). In other systems, FLV upregulates IL-10 (Kalkman and Feuerbach, 2016; Nazimek et al., 2017). FLV via the S1R may therefore modulate SARS-CoV-2-induced hyperinflammatory state (Figure 1).

On the flip side, genetic perturbation screens have shown depletion of S1R, *decreases* SARS-CoV-2 viral replication in adenocarcinoma human alveolar basal epithelial cell lines expressing Angiotensin I Converting Enzyme 2 (A549-ACE2) (Gordon et al., 2020). Consistent with this genetic data, S1R agonists such as dextromethorphan can increase viral replication (Gordon et al., 2020). However, in contrast, researchers reviewing medical billing data for nearly 740,000 COVID-19 patients in the US showed patients on antipsychotic drugs targeting S1R were half as likely as those on other types of antipsychotic drugs to require mechanical ventilation (Gordon et al., 2020).

Neurotropism is one common feature for human coronaviruses (Bale, 2015; Dube et al., 2018). Various receptors could be involved in neurotropism and neuronal cell entry of SARS-CoV-2 (Armocida et al., 2020). Sigma receptors are widely expressed in the CNS (Yesilkaya et al., 2020). Downregulation of S1R protein expression impairs initiation of hepatitis C virus (HCV) RNA replication in human hepatoma cells (Friesland et al., 2013). BD1047 a selective S1R antagonist blocked cocaine-mediated stimulation of human immune deficiency virus (HIV-1) expression in neuronal mononuclear phagocytes like microglia (Gekker et al., 2006). S1R could therefore be involved in neuronal transmission of other RNA viruses like SARS-CoV-2.

### Inositol-Requiring Enzyme 1α and Autophagy

Endotoxin-stimulated TLR4 activates IRE1 (Martinon et al., 2010) and regulates proinflammatory cytokine production (Qiu et al., 2013). SARS-CoV E protein down-regulates IRE1 pathway and the SARS-CoV lacking the envelope (E) gene (rSARS-CoV-ΔE) is attenuated *in vivo* (DeDiego et al., 2011). IRE1 inhibitors like STF-083010 rescued S1R KO mice in a model of endotoxemia (Rosen et al., 2019). IRE1 is essential for autophagy during infection with a gamma coronavirus-Infectious Bronchitis Virus (IBV) (Fung and Liu, 2019). SARS-CoV replicase proteins nsp2, 3 and 8 occur in cytoplasmic complexes and colocalize with LC3, a protein marker for autophagic vacuoles (Prentice et al., 2004). The viral replicase protein nsp6 of IBV activates autophagy in a screen (Cottam et al., 2011). Other studies reviewed here (Yang and Shen, 2020) suggest autophagy is not directly involved in SARS-CoV. These discrepancies are probably because of different viruses and cells tested in various studies.

### Melatonin

SARS-CoV-2 virus can activate NLRP3 inflammasome (van den Berg and Te Velde, 2020), which along with NF-κB activation can induce cytokine storm (Ratajczak and Kucia, 2020). Melatonin can mitigate inflammation through these pathways and melatonin exposure post-intubation is associated with a positive outcome in COVID-19 (and non-COVID-19) patients (Garcia et al., 2015; Ramlall et al., 2020). FLV can elevate melatonin levels via inhibition of CYP1A2, a member of the cytochrome P450 superfamily of enzymes (Hartter et al., 2001) (Figure 1).

## Could Selective Serotonin Reuptake Inhibitors and Sigma-1 Receptor Agonists Have Direct Antiviral Effects on Other Viruses?

### Precedent for Using Selective Serotonin Reuptake Inhibitors to Treat Other Viral Infections

Enteroviruses are non-enveloped RNA viruses. Their nonstructural protein 2C is one of their most conserved proteins and contains ATPase activity and putative RNA helicase activity (Cheng et al., 2013). Fluoxetine has *in vitro* antiviral activity against *Enterovirus B* and *D* species (Zuo et al., 2012; Ulferts et al., 2013). Fluoxetine binds nonstructural protein 2C directly (Manganaro et al., 2020). Some fluoxetine resistant variants of enteroviruses like coxsackievirus B3 and B4 have mutations in protein 2C (Ulferts et al., 2013; Alidjinou et al., 2019). This reinforces the idea that interaction between fluoxetine and protein 2C is essential for its antiviral effects.

### Endoplasmic Reticulum Stress Response

Viral infection may trigger the unfolded protein response (UPR). This is an ER stress response because of ER overloading with virus-encoded proteins (Kim et al., 2008), and can also induce autophagy (Bernales et al., 2006; Ogata et al., 2006). ER signaling proteins like IRE1, PRKR-like ER kinase (PERK), and activating transcription factor 6 (ATF6) regulate UPR. The UPR is involved in viral replication and modulates host innate responses (Xue et al., 2018). Virus-induced ER stress is required for autophagy activation, viral replication, and pathogenesis in dengue (Lee et al., 2018). Murine cytomegalovirus activates the IRE1 pathway to relieve repression by X-box binding protein 1 unspliced mRNA (Hinte et al., 2020). Coronavirus infection induces ER stress and triggers UPR (Fung et al., 2016). The S protein in β-coronaviruses modulates UPR to facilitate viral replication (Chan et al., 2006; Versteeg et al., 2007). The α-coronavirus, transmissible gastroenteritis virus (TGEV) triggers UPR-induced ER stress primarily through activation of PERK-eukaryotic initiation factor 2α axis (Xue et al., 2018). Thus ER stress response is critical in host-virus interactions in a variety of infections. We have discussed above how S1R is a regulator of IRE1 and autophagy. S1R agonists like FLV could therefore have a role in regulating viral infections beyond SARS-CoV-2 through its putative regulation of ER stress and UPR.

## Preclinical Effects of Fluvoxamine on Inflammation

S1R KO mice display increased mortality compared to WT in sublethal models of sepsis (Rosen et al., 2019). Peak serum TNF and IL-6 were increased in LPS-challenged S1R KO mice. S1R ligand FLV enhanced survival in mouse models of IRE1-mediated inflammation and fecal-induced peritonitis. FLV treatment protected WT mice from endotoxic shock-induced death, while no significant effect was observed in S1R KO animals suggesting the anti-inflammatory effects of FLV are likely mediated through S1R.

Multiple sclerosis (MS) is a chronic, inflammatory, demyelinating neurodegenerative disease. SSRIs like sertraline have been shown to have immunomodulatory effects in experimental autoimmune encephalomyelitis (EAE), a mouse model of MS (Taler et al., 2011), and in a rat model of rheumatoid arthritis (Baharav et al., 2012). FLV reduces the severity in EAE in rats, even when treatment began 12 days post-induction of EAE (Ghareghani et al., 2017). FLV-treated EAE rats showed a decrease in IFN-γ serum levels and an increase in IL-4, pro- and anti-inflammatory cytokines respectively, compared to untreated EAE rats. The dose of FLV used in these experiments extrapolates (by surface area) to FLV doses approved for human use.

Thus, FLV seems to ameliorate inflammation in different *in vivo* inflammation models. Data in non-human primates or a hamster model of SARS-CoV-2 infection would shed further light on whether FLV might be a useful drug for COVID-19 patients and on the mechanism(s) at play.

## Clinical Effects of Fluvoxamine in COVID-19

In a double-blind, randomized, preliminary study of adult outpatients with symptomatic COVID-19, 80 patients treated with FLV, compared to 72 treated with placebo, had a lower likelihood of clinical deterioration over 15 days (Lenze et al., 2020). Eligible patients were enrolled within 7 days of symptom development. These data are provocative with none of the FLV-treated patients deteriorating vs. 8.3% patients in the control arm who showed clinical deterioration. Participants received 50 mg FLV QD on day 1, then for 2 days 100 mg FLV BID, and then 100 mg FLV TID as tolerated through day 15 and then stopped. In a prospective study on use of FLV for early treatment of COVID-19 the incidence of hospitalization was 0% (0/65) with FLV and 12.5% (6/48) with observation alone. At 14 days, 0% (0/65) of FLV treated people had persistent residual symptoms compared to 60% (29/48) among people who opted for no therapy (Seftel and Boulware, 2021). Agonists of S1R like escitalopram and fluoxetine were associated with lower risk of intubation or death (*p* < 0.05) because of COVID-19 in a multicenter observational retrospective cohort study (Hoertel et al., 2021).

Given the multiple roles of S1R reviewed here in inflammation, platelet aggregation, antiviral activity etc. and the recent striking human data, it is likely that S1R agonists like FLV could have a major impact on disease progression of COVID-19 patients in the early stage of the disease.

## Discussion

An 880 patient randomized study is underway and should provide some definitive answers (Lenze, 2020). Patients nationally can join this study from home and at no cost. However, *given the current crisis*, which is expected to worsen before a vaccine takes effect, one wonders if the FLV evidence in COVID-19 is strong enough to consider a change in practice guidelines, to even more quickly accumulate data on outcomes in COVID-19 patients (Sukhatme and Sukhatme, 2021). A small group of healthcare systems could consider this approach and simultaneously set up tools, e.g., a local or regional repository to track outcomes in real-time. If the efficacy is similar to the small randomized trial (Lenze et al., 2020), it should be evident in such data. Out of caution, the practice guidelines could urge caregivers to consider administering FLV only to those COVID-19 + patients at highest risk for disease progression, and who do not have access to one of the monoclonal antibodies that have been given emergency use authorizations by the FDA (FDA, 2020a; FDA, 2020b). Also, these guidelines could be revised at any time.

Small biomarker intensive trials should be planned to assess antiviral, immunomodulatory, anti-thrombotic effects or other effects in patients treated with FLV. One could incorporate tools such as single cell RNA and protein analysis in such studies. While human data is being gathered, additional preclinical data in cell culture systems like co-cultures of human epithelial and immune cells would be useful (Grunwell et al., 2019). Data from non-human primate and hamsters would provide valuable information on optimal timing of drug, amount needed for efficacy, and which among the myriad mechanisms of action might be most relevant.

There may be a role for serotonin modulation in the inpatient setting. Indeed, if this drug is not working primarily as an antiviral but rather through other mechanisms (e.g., immunomodulatory, anti-platelet), it may be efficacious in this setting where hyperinflammatory responses and thrombotic events drive disease pathology. However, there will need to be vigilance for emergence of a hyperserotonergic state with similarities to serotonin syndrome, as noted earlier. Thus it may make sense to initiate fluvoxamine in the less severe hospitalized patients but administer a serotonin 2 A, B and C receptor antagonist such as cyproheptadine or mirtazapine in the more severe patients (along with fluvoxamine). It is also tempting to speculate on a role for FLV in COVID-19 long-haulers. There are likely to be subsets in this heterogeneous group that may have an aberrant immune response that has lingered on, in which FLV may be efficacious. Finally, there may be a role for FLV in the treatment of other viral illnesses in which there is some version of a cytokine storm present (Fajgenbaum and June, 2020).
